# Perspectives on Telemedicine Visits Reported by Patients With Cancer

**DOI:** 10.1001/jamanetworkopen.2024.45363

**Published:** 2024-11-15

**Authors:** Sahil D. Doshi, Yasin Khadem Charvadeh, Kenneth Seier, Erin M. Bange, Bobby Daly, Allison Lipitz-Snyderman, Fernanda C. G. Polubriaginof, Michael Buckley, Gilad Kuperman, Peter D. Stetson, Deb Schrag, Michael J. Morris, Katherine S. Panageas

**Affiliations:** 1 Memorial Sloan Kettering Cancer Center, New York, New York; 2 Weill Cornell Medical College, New York, New York

## Abstract

**Importance:**

The COVID-19 pandemic catalyzed rapid adoption of telemedicine visits for cancer care delivery. However, patients’ experiences with telemedicine remain poorly understood.

**Objective:**

To understand patients’ satisfaction with telemedicine visits at a comprehensive cancer center.

**Design, Setting, and Participants:**

This survey study included patients with cancer at a US cancer center between 2020 and 2023. Eligible patients completed surveys in English conducted after their first telemedicine appointment via an online patient portal. Data were analyzed between January and June 2024.

**Exposures:**

Patient surveys about telemedicine experiences, with a specific comparison with an in-person visit. Structured items elicited satisfaction with the specific visit, preferences for future use of telemedicine, and technical ease of use; unstructured free-text responses were also elicited.

**Main Outcomes and Measures:**

Proportion of patients who indicated that telemedicine visits were superior or preferred to in-person visits. Secondary outcomes included multivariable analysis of barriers to telemedicine use and variations in patient experiences by demographic characteristics over the observation period, and free-text analysis of unstructured responses describing the telemedicine experience using the BERTopic algorithm and a language model.

**Results:**

A total of 27 435 telemedicine users completed surveys from May 2020 to October 2023 (median [IQR] age, 65 [55-72] years; 15 072 female [54.9%]; 1771 Asian [6.7%], 1339 Black [5.1%], 22 742 White [85.9%]). Overall, 18 025 of 24 418 patients (73.8%) rated their first telemedicine visit as good as or better than an in-person visit, and 4606 (18.9%) rated it superior to an in-person visit. The proportion of patients rating a telemedicine visit superior to an in-person visit evolved from 17% in 2020 to 20% in 2023. Structured questions revealed a positive view of telemedicine, while free-text analyses highlighted issues with technology.

**Conclusions and Relevance:**

In this survey study of perspectives on telemedicine visits, a large majority of patients at a comprehensive cancer center expressed satisfaction with telemedicine visits in proportions that remained consistent beyond the end of the pandemic. These findings challenge health care systems to integrate telemedicine into routine cancer care and to overcome remaining technical challenges and barriers to ease of use.

## Key Points

**Question:**

Are patients with cancer satisfied with telemedicine visits?

**Findings:**

This survey study of 27 435 patients with cancer treated at a single US cancer center between 2020 and 2023 identified high levels of satisfaction with telemedicine visits, desire for future telemedicine visits, and stable annual trends over time. Topic modeling analysis of patient free-text responses highlighted ongoing issues with telemedicine that need improvement.

**Meaning:**

These results suggest that patients report high satisfaction rates with telemedicine, challenging clinicians and health care systems to implement strategies to optimize care delivery using telemedicine.

## Teaser

This survey study of patients with cancer treated at a US cancer center between 2020 and 2023 examines patient satisfaction following their first telemedicine visit relative to in-person visits and describes areas of potential improvement.

## Introduction

The growing time and cost burdens of cancer care on patients, health care professionals, and systems has led to a focus on optimizing accessibility and value. Once sparingly utilized, telemedicine surged during the COVID-19 pandemic to maintain care accessibility amidst safety concerns. Telemedicine, defined as the use of electronic information and communication to deliver health care remotely, has since emerged as an approach to ensuring accessibility.^1^ Despite satisfaction with telemedicine as a coping strategy during the pandemic, subsequent uncertainty about its effectiveness and the policies governing reimbursement make the future role of telemedicine uncertain.

Telemedicine was infrequently used in cancer care prior to the COVID-19 pandemic, accounting for fewer than 1% of all Medicare outpatient encounters.^2^ Prepandemic telemedicine use was confined to specific scenarios including care in rural communities, genetic counseling, and for survivorship.^3,4,5,6,7,8,9,10,11,12^ The pandemic dramatically altered this landscape, and a transformed regulatory environment facilitated the broad, rapid adoption of telemedicine use. For example, in quarter 2 of 2020 at the height of the pandemic, 47% of all Medicare users had at least 1 telemedicine service compared with 7% in quarter 1.^13^

Although feasibility and generally high rates of satisfaction with telemedicine became evident during the pandemic, the role of telemedicine in health care now remains uncertain, and satisfaction since the early stages of the COVID-19 pandemic remains largely unexplored.^14,15,16,17,18^ This is especially true for patients with cancer who have complex chronic needs. On the one hand, telemedicine may support accessibility, convenience, and contain costs. On the other hand, it may hinder effectiveness or undermine safety if lost opportunities for physical examination prevent timely identification of treatment complications or manifestations of cancer progression.

We sought to understand the experience of patients with cancer with telemedicine by surveying patients attending their first telemedicine visit at a single specialty center from the height of the pandemic through its resolution. We evaluated patients’ perceptions of telemedicine visits compared with in-person visits, the likelihood of recommending a telemedicine visit to another patient, and their preferences for future telemedicine visits using structured surveys. In addition, we explored perceptions by inviting free-text responses eliciting extensive and detailed patient insights of the successes and challenges of telemedicine. Insights into patient satisfaction in these settings can lead to key improvements and support the appropriate adoption and integration of telemedicine into health care practices.

## Methods

### Study Design and Participants

We surveyed all first-time telemedicine users at Memorial Sloan Kettering (MSK) Cancer Center between May 2020 to October 2023. Adult patients who had their initial telemedicine visit with a physician or nurse practitioner in any specialty received a survey in English through MSK’s patient portal. Patients were required to have portal enrollment to receive the survey. All participants had at least 1 previous in-person visit with an MSK clinician. Patients received surveys after visits to any MSK health care professional, such as surgeons and medical and radiation oncologists as well as other specialties such as cardiologists, neurologists, and psychiatrists. Baseline patient characteristics including age, sex, self-reported race or ethnicity (American Indian or Alaska Native, Asian, Black or African American, Native Hawaiian or Pacific Islander, White, and other), and state of residence were collected from the electronic health record. Race and ethnicity were considered as they have been shown to be a relevant variable for satisfaction with telemedicine. The study was approved by the Memorial Sloan Kettering Cancer Center institutional review board on February 20, 2023, and patients were not required to provide informed consent because our study involved minimal risk and used limited identifiable information. This report follows the American Association for Public Opinion Research (AAPOR) reporting guideline for survey studies.

### Questionnaire Content

The questionnaire comprised 10 structured questions about the telemedicine experience and 10 questions regarding clinical trial education and informed consent. Seven of the first 10 structured items were followed by an option for a free text comment (eAppendix in Supplement 1). Additionally, a comment box was provided for patients to share any further thoughts or feelings about the informed consent process. In this study, we focused on the first 10 questions about the telemedicine experience and all the free-text comments.

Patients were asked, “How did having a telemedicine visit compare with having an in-person visit with your health care provider?” The primary outcome was the proportion of patients who indicated that a telemedicine visit was superior to or preferred to an in-person visit. Other questions focused on the technical aspects of the telemedicine visit, including device usability and instructions on how to connect, and general satisfaction, including whether a patient would have another telemedicine visit and if they would recommend it to other patients. The full survey is available in eAppendix in Supplement 1.

### Statistical Analysis

#### Structured Survey Questions

Categorical variables were described using count and percentage, and continuous variables were described using median and interquartile range (IQR). We quantified patient responses for each survey question and stratified responses by 1-year intervals between May 2020 to October 2023. Demographic characteristics were compared between response categories for the question, “How did having a telemedicine visit compare with having an in-person visit with your health care provider?” using a χ^2^ test or Fisher exact test for categorical variables and a Wilcoxon rank sum test for continuous variables. R version 4.3.3 (R Foundation for Statistical Computing) and Python version 3.12.0 (Python Software Foundation) were used for all analyses. All tests were 2-sided and *P* < .05 was considered significant.

#### Topic and Language Modeling

Analysis of structured responses provided a foundational overview of patient preferences and perceived barriers to telemedicine. However, relying solely on structured data limits our ability to capture the nuances of patient experiences and concerns, as these responses offer predefined options that may not fully encompass patient sentiments and detailed feedback. In contrast, unstructured responses allow patients to articulate their experiences in their own words, revealing details and insights that structured approaches might miss. Therefore, integrating unstructured data into our analysis was essential for capturing the richness and complexity of patient perspectives on telemedicine.

To analyze the unstructured free-text responses, we employed BERTopic, an advanced topic modeling algorithm that leveraged transformer-based embeddings to facilitate the creation of easily interpretable topics from a collection of text documents.^19^ Through topic modeling with BERT (Bidirectional Encoder Representations from Transformers) embeddings, we can capture nuanced language patterns and semantic relationships, thus enhancing the identification of meaningful topics. It should be noted that all free-text responses were analyzed as a single variable and not within a structured question.

The BERTopic algorithm produced several key outputs that facilitate a comprehensive understanding of the topics identified within a document corpus. One primary output is the list of topics, each characterized by a set of representative keywords known as *topic representation*. These keywords are the most indicative terms for each topic, helping to succinctly define the topic’s essence. Additionally, the algorithm outputs the frequency of each topic across the entire corpus, revealing how prevalent each topic is. This frequency count helps in understanding the distribution and dominance of topics within the corpus.

The algorithm also provides document-topic probabilities, indicating the strength of association between each document and the identified topics. Furthermore, the BERTopic algorithm identifies representative documents for each topic, selecting those that best exemplify the topic based on their high association scores. Together, these outputs offer a detailed view of the topics, their relevance, and their semantic relationships, enabling effective analysis and interpretation of large sets of textual data.

BERTopic assumes each document is associated with only 1 topic, but documents often contain multiple topics. To address this, we used a token-level approach with a sliding window of size 4, as recommended by BERTopic’s author.^20^ Testing different window sizes (6 and 8) increased outlier documents without significantly changing the frequency of most identified topics. To maintain a comparable number of outliers with the single-topic approach, we set a probability threshold of 0.15 for topic attribution. Additionally, we counted unique medical record numbers per topic to correct for repeated comments, offering a more accurate topic prevalence.

While topic modeling provides a basic understanding of discussed topics and their relative prominence, it may not provide insight into specific details or sentiments related to various aspects of the telemedicine visits. Interpreting the topics identified can be challenging and often necessitates domain knowledge or a contextual understanding of the data. To gain deeper insights into the identified topics and their related documents, we utilized Mistral 7B–Instruct, a 7-billion-parameter language model (LM) that has demonstrated its superiority over certain high-level LMs across various aspects.^21^ Specifically, we used this LM to summarize the comments associated with the identified topics. This is achieved by feeding the model with comments corresponding to each topic, along with a specific instruction. The model processes this input and produces comprehensive summaries, shedding light on the nuances of the patient discourse.

## Results

### Structured Survey Question Results

A total of 27 435 first-time users of telemedicine completed questionnaires between May 2020 and October 2023 (median [IQR] age, 65 [55-72] years; 15 072 female [54.9%]; 1771 Asian [6.7%], 1339 Black [5.1%], 22 742 White [85.9%]) (Table). Across the study years, 16 240 surveys were completed in 2020; subsequent years had 5347 respondents from 2021, 4274 from 2022, and 1574 from 2023. The number of respondents declined over time because surveys were only sent to patients following their first telemedicine visit to an MSK physician or advanced practice clinician.

**Table.** Patient Characteristics According to Their Perceptions of Telemedicine Visits

Characteristic	Patients, No. (%)					*P* value^b^
	Total patients (n = 27 435)^a^	Having a televisit was about the same as an in-person visit (n = 13 419)	Having a televisit was better than an in-person visit (n = 4606)	Having a televisit was not as good as an in-person visit (n = 6393)	Not sure (n = 2801)	
Age at encounter, median (IQR), y	65 (55-72)	64 (54-72)	63 (54-71)	67 (58-74)	64 (55-72)	<.001
Sex						
Female	15 072 (54.9)	7394 (55.1)	2449 (53.2)	3383 (52.9)	1720 (61.4)	<.001
Male	12 363 (45.1)	6025 (44.9)	2157 (46.8)	3010 (47.1)	1081 (38.6)	
Race						
American Indian or Alaska Native	22 (<0.1)	6 (<0.1)	5 (0.1)	7 (0.1)	3 (0.1)	
Asian	1771 (6.7)	832 (6.4)	341 (7.7)	320 (5.2)	265 (9.9)	<.001
Black or African American	1339 (5.1)	715 (5.5)	215 (4.8)	251 (4.1)	145 (5.4)	
Native Hawaiian or Pacific Islander	16 (<0.1)	5 (<0.1)	4 (<0.1)	4 (<0.1)	3 (0.1)	
White	22 742 (85.9)	11 124 (85.7)	3776 (85.0)	5489 (88.9)	2173 (81.2)	
Other	586 (2.2)	296 (2.3)	99 (2.2)	102 (1.7)	87 (3.3)	
Unknown	959	441	166	220	125	
Ethnicity						
Hispanic or Latino	1503 (5.8)	758 (6.0)	283 (6.5)	255 (4.2)	194 (7.3)	<.001
Not Hispanic or Latino	24 548 (94.2)	11 972 (94.0)	4093 (93.5)	5840 (95.8)	2454 (92.7)	
Unknown	1384	689	230	298	153	
Preferred language						
English	26 732 (97.5)	13 077 (97.6)	4463 (97.0)	6295 (98.6)	2689 (96.1)	<.001
Other	674 (2.5)	326 (2.4)	140 (3.0)	92 (1.4)	108 (3.9)	
Unknown	29	16	3	6	4	
Marital status						
Divorced	1678 (6.2)	818 (6.2)	275 (6.0)	382 (6.0)	192 (7.0%)	.056
Married	19 588 (72.2)	9660 (72.7)	3278 (72.1)	4599 (72.6)	1895 (68.8)	
Partnered	294 (1.1)	128 (1.0)	50 (1.1)	78 (1.2)	38 (1.4)	
Separated	228 (0.8)	111 (0.8)	37 (0.8)	52 (0.8)	25 (0.9)	
Single	4008 (14.8)	1945 (14.6)	676 (14.9)	900 (14.2)	461 (16.7)	
Widowed	1343 (4.9)	628 (4.7)	232 (5.1)	325 (5.1)	142 (5.2)	
Unknown	296	129	58	57	48	
State of residence						
Connecticut	1171 (4.3)	578 (4.3)	225 (4.9)	274 (4.3)	84 (3.0)	<.001
Florida	967 (3.5)	468 (3.5)	160 (3.5)	269 (4.2)	64 (2.3)	
New Jersey	7109 (25.9)	3544 (26.4)	1224 (26.6)	1573 (24.6)	723 (25.8)	
New York	15 788 (57.5)	7619 (57)	2567 (55.7)	3750 (58.7)	1712 (61.1)	
Other	2400 (8.7)	1210 (9.0)	430 (9.3)	527 (8.2)	218 (7.8)	
Telemedicine visit year						
2020	16 240 (59.2)	7608 (56.7)	2414 (52.4)	4501 (70.4)	1598 (57.0)	<.001
2021	5347 (19.5)	2691 (20.1)	1055 (22.9)	970 (15.2)	580 (20.7)	
2022	4274 (15.6)	2262 (16.9)	855 (18.6)	657 (10.3)	473 (16.9)	
2023	1574 (5.7)	858 (6.4)	282 (6.1)	265 (4.1)	150 (5.4)	

^a^ 216 patients did not answer the question of in-person versus telemedicine preference.

^b^ Kruskal-Wallis rank sum test, Pearson χ^2^ test, Fisher exact test for count data with simulated *P* value (based on 2000 replicates).

**Figure 1.**
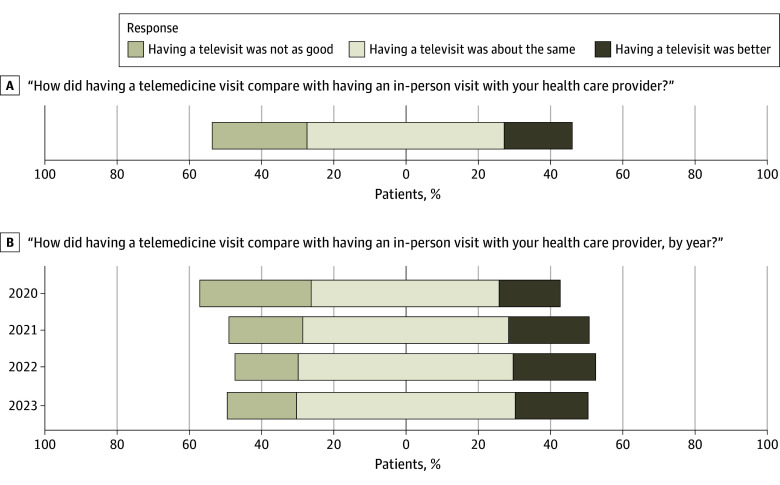
Satisfaction With Telemedicine Visits Compared With In-Person Visits

Excluding 2801 patients who answered “not sure,” a total of 18 025 of 24 418 patients (73.8%) rated their first telemedicine visit at MSK as good as or better than an in-person visit, and 4606 of 24 418 (18.9%) rated it superior to an in-person visit (Figure 1A). When this response was evaluated by year, we found that patient satisfaction with telemedicine was lowest in 2020, with 4501 of 14 523 (31.0%) noting that a telemedicine visit was not as good as an in-person visit; rates have since stabilized from 2021 to 2023 to 265 of 1405 patients (18.9%) noting that a telemedicine visit was inferior (Figure 1B). Satisfaction levels were consistently high with small variation based on patient characteristics (Table). Patients responding, “Having a televisit was not as good” were older on average (median [IQR] age: not as good, 67 [58-74] years vs same, 64 [54-72] years vs better, 63 [54-71] years; *P* < .001). With regards to race, White patients were slightly less satisfied with telemedicine compared with in-person, Asian patients were slightly more satisfied with telemedicine, and Black patients found it about the same (Asian: not as good, 320 of 6173 patients [5.2%] vs same, 832 of 12 978 patients [6.4%] vs better, 341 of 4440 [7.7%]; Black: not as good, 251 of 6173 patients [4.1%] vs same, 715 of 12 978 patients [5.5%] vs better, 215 of 4440 patients [4.8%]; White: not as good, 5489 of 6173 patients [88.9%] vs same, 11 124 of 12 978 patients [85.7%] vs better, 3776 of 4440 [85.0%]; *P* < .001). The percentages reflect the distribution within each Likert scale response.

**Figure 2.**
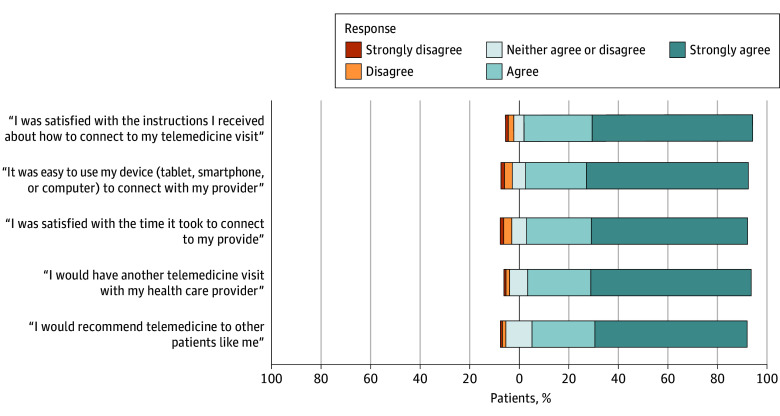
Satisfaction With Telemedicine Visits Reported by Outpatients With Cancer

**Figure 3.**
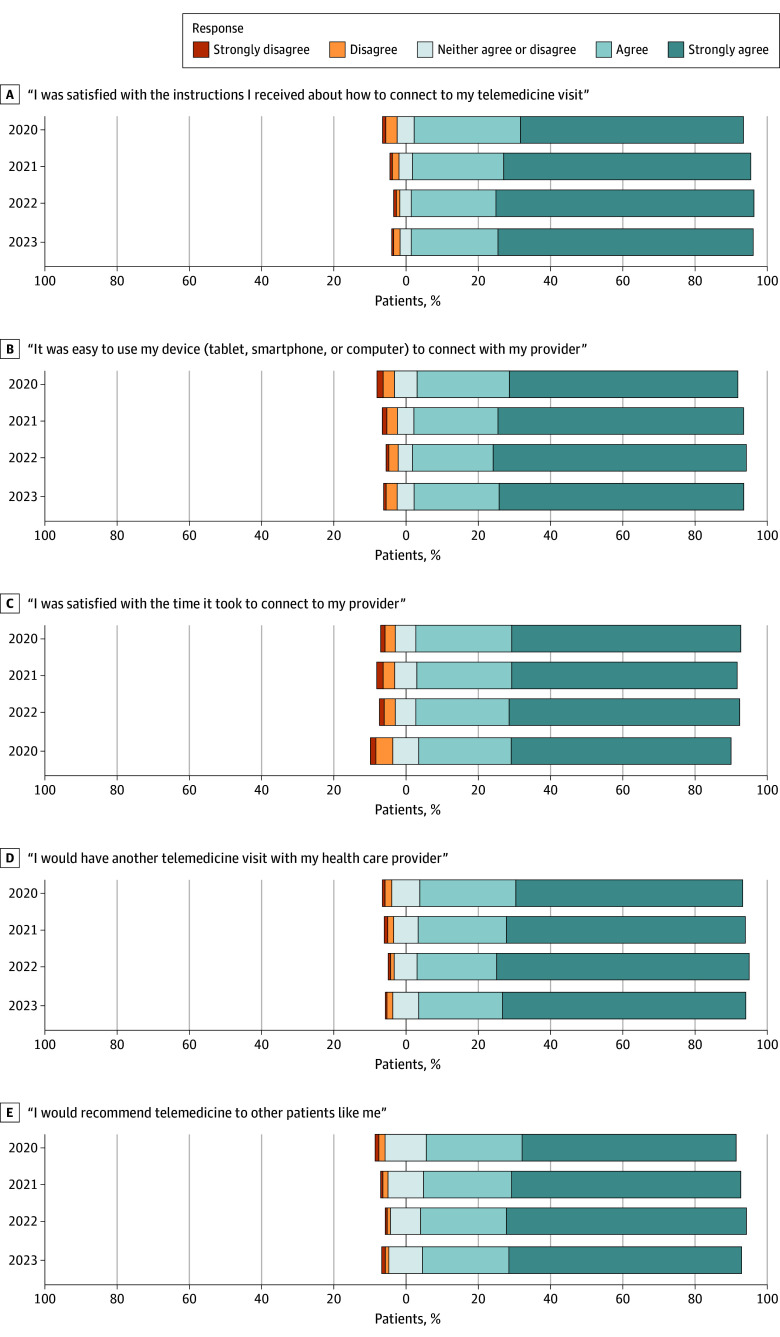
Satisfaction With Telemedicine Reported by Outpatients With Cancer by Year

Overall satisfaction levels with other survey questions were high, with 24 602 of 27 254 patients (90.3%) expressing preference for future telemedicine visits and 23 769 of 27 278 patients (87.1%) recommending telemedicine to other patients (Figure 2). Most patients were also satisfied with the connection process, with 24 837 of 27 386 patients (90.7%) expressing satisfaction with the instructions they received and 24 543 of 27 209 patients (90.2%) expressing ease with using their device of choice (computer, tablet, or smartphone) to connect with their clinician. These responses have remained largely consistent across 2020 through 2023 (Figure 3).

### Free-Text Survey Results: Insights From Topic and Language Modeling

Overall, 8418 of 27 435 respondents (30.7%) contributed at least 1 comment, with some leaving multiple comments. Implementing BERTopic, we identified 37 topics within the unstructured dataset. Key topics included comments on delayed appointments and scheduling systems, issues with video or audio functionality, preference for in-person or virtual visits, necessity of physical examination, clear or unclear instructions, connection problems, staff helpfulness, having a voice call, device compatibility, doctor-patient interaction, web browser issues and incompatibility, digital literacy, and friends and family invitation feature of the telemedicine platform. The value of the topic modeling lay in revealing issues the structured questions could not address and highlighting the prominence of common issues and concerns. To illustrate the prominence of each topic, we generated a topic cloud offering a visual representation of the frequency of the key topics (eFigure 1 in Supplement 1). The topic cloud highlights frequent comments on appointment delays, preferences for visit types, the necessity of physical examinations, connection issues, device compatibility, and having a voice call instead of a telemedicine visit.

To better understand the essence of the identified key topics and their potential semantic relationships, we created a co-occurrence network (eFigure 2 in Supplement 1). This figure illustrates the top keywords for each key topic, providing a view of the most distinctive terms associated with each theme. For example, the topic device compatibility includes keywords such as *laptop*, *iPhone*, *smartphone*, *apple*, and *devices*. Moreover, some topics show interconnectedness through shared keywords. Topics such as clear or unclear instructions, issues with video or audio functionality, digital literacy, web browser issues and incompatibility, device compatibility, and connection problems demonstrate thematic connections through common keywords. Conversely, topics like friends and family invitation feature of the telemedicine platform and staff helpfulness remain distinct, sharing no overlap with other topics. The presence of shared keywords suggests potential causal relationships between topics. For example, the topics device compatibility and connection problems are linked through the keyword *connect*, indicating that device incompatibility may lead to connection issues.

**Figure 4.**
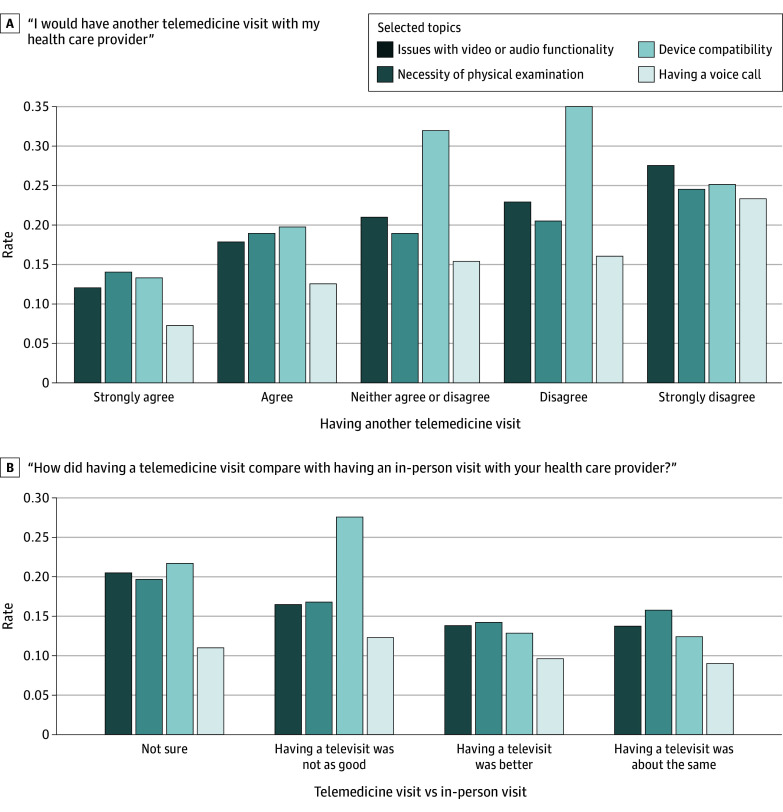
Rates of Selected Topic Occurrences With Survey Questions

To understand if there were any associations between some of the key topics and patients’ responses to certain questions in the survey, we plotted the rate of topic occurrences within the distinct Likert scales associated with 2 survey questions (Figure 4). The patterns suggest that patients mentioning topics associated with negative sentiments toward telemedicine—such as issues with video or audio functionality, necessity of physical examination, device compatibility, and having a voice call—were less likely to prefer or choose another telemedicine visit.

Summaries of comments associated with each of the key topics, generated using the Mistral 7B–Instruct model, are reported in the eTable in Supplement 1. These summaries highlight a mix of positive and negative experiences. While some patients appreciated the convenience and time savings of telemedicine appointments, others faced technical difficulties and preferred in-person visits due to the importance of physical examinations and personal connection.

## Discussion

Our analysis of 27 435 patients treated at a large urban academic cancer center revealed high satisfaction of first-time users of cancer care by telemedicine that has persisted since the initial surge in 2020. Our survey also included a rich opportunity for free-text responses from patients. With topic modeling using BERTopic, we were able to effectively analyze these free-text responses and glean additional insight to add to the structured question results, an opportunity which is often underutilized in survey-based studies. We effectively identified the prevalence of certain issues and concerns with telemedicine including technical challenges and limitations with the physical examination. Language modeling summaries highlighted recurring themes in detail and areas ripe for improvement in telemedicine delivery.

Our study importantly highlights that patient satisfaction with telemedicine remains high in recent years. While most survey respondents were from 2020 to 2021, 5848 first-time telemedicine users at MSK completed the questionnaire between 2022 and 2023, with a large majority indicating high satisfaction and willingness for future telemedicine visits. The survey focused only on first-time telemedicine users, explaining the decrease in respondents over time as more patients gained prior telemedicine exposure at MSK. Satisfaction with telemedicine was lowest in 2020 but has stabilized from 2021 to 2023, likely due to early technical challenges that have since improved. However, impending national policy changes could complicate the provision of telemedicine-based cancer care for patients who prefer and benefit from it. Analyzing changes in patient satisfaction over time can inform policymakers as they refine telemedicine regulations and support systems to make telemedicine more appealing and effective for clinicians and patients.

At the start of the COVID-19 pandemic, the Centers for Medicare and Medicaid Services introduced regulations that expanded telemedicine adoption. As the pandemic ends, many of these changes are being phased out, posing barriers to telemedicine in cancer care. Medicare waivers expiring in December 2024 include allowing patients to receive telemedicine services at home and in any geographic area.^22^ This expiration, although extended through 2026 pending Congress approval, raises concerns about telemedicine’s future, despite patients’ high satisfaction and preferences.^23^ Additionally, the rescission of state licensure requirement waivers has made providing telemedicine services across state borders difficult. Significant regulatory efforts are therefore needed to maintain telemedicine’s role in cancer care.

Although our findings demonstrated high rates of satisfaction across years and patient subgroups, there were several themes that emerged regarding ongoing challenges with telemedicine use. Our free-text analyses indicated that certain patients struggle with the technical aspects of a telemedicine visit, including video and audio functionality, device compatibility, and web browser issues. Furthermore, certain patients commented on the limited physical examination capabilities via telemedicine. Patients who commented on these challenges and limitations with telemedicine were more likely to note that they preferred in-person visits. These results highlight that certain patients may benefit from digital navigation and training to better facilitate their care experience.

### Limitations

This study had several limitations. Our results are limited by the patient cohort and data availability; although we had a large number of patient respondents, the analysis is limited to a single cancer center, and therefore generalizability is restricted. Furthermore, we had limited access to baseline clinical characteristics from patients including visit type and cancer types, preventing us from exploring how different points in the cancer treatment continuum might affect satisfaction rates. Additionally, our study was limited to patients who utilized the patient portal to complete the survey, potentially missing those with telemedicine visits who did not use the portal, and we do not have information on the patients who did not complete the survey.

## Conclusions

In this survey study of patient perspectives on telemedicine, we found that patient satisfaction with telemedicine in cancer care delivery remained high after the COVID-19 pandemic. It is therefore important to support telemedicine at individual, institution, state, and federal levels, and identify best practices for those patients who benefit most from it. By employing novel methods like BERTopic modeling to scrutinize patient feedback in detail, health care professionals can pinpoint the most valued aspects of telemedicine and areas needing enhancement, leading to more user-friendly services that align closely with patient needs. Furthermore, the pandemic demonstrated telemedicine’s capacity to maintain continuity of care during disruptions. Ongoing monitoring and enhancement of patient satisfaction can sustain and expand telemedicine use, making health care more accessible and efficient. For patients with cancer, effective telemedicine can offer significant benefits by decreasing in-person visits, reducing exposure to infections, and improving treatment adherence. Ongoing research ensures that the future role of telemedicine is evidence-based, capturing patient experiences and satisfaction, including in the postpandemic era.

## Notes

Supplement 1. **eAppendix.** Survey Questions **eFigure 1.** Topic Cloud of the Key Topics **eFigure 2.** Top Keywords Associated with Key Topics **eTable.** Key Topics, Representative Comments, and Language Modeling Summary

Supplement 2.
Data Sharing Statement

## References

[1] Institute of Medicine (US) Committee on Evaluating Clinical Applications of TM. A Guide to Assessing Telecommunications for Health Care. The National Academies Press; 1996. 20845554

[2] Patel SY, Mehrotra A, Huskamp HA, Uscher-Pines L, Ganguli I, Barnett ML. Trends in outpatient care delivery and telemedicine during the COVID-19 pandemic in the US. JAMA Intern Med. 2021;181(3):388-391. doi:10.1001/jamainternmed.2020.5928 33196765 PMC7670397

[3] Doolittle GC, Spaulding AO. Providing access to oncology care for rural patients via telemedicine. J Oncol Pract. 2006;2(5):228-230. doi:10.1200/jop.2006.2.5.228 20859340 PMC2793628

[4] Danylchuk NR, Cook L, Shane-Carson KP, . Telehealth for genetic counseling: a systematic evidence review. J Genet Couns. 2021;30(5):1361-1378. doi:10.1002/jgc4.1481 34355839

[5] Bradbury A, Patrick-Miller L, Harris D, . Utilizing remote real-time videoconferencing to expand access to cancer genetic services in community practices: a multicenter feasibility study. J Med Internet Res. 2016;18(2):e23. doi:10.2196/jmir.4564 26831751 PMC4754531

[6] Buchanan AH, Datta SK, Skinner CS, . Randomized trial of telegenetics vs in-person cancer genetic counseling: cost, patient satisfaction and attendance. J Genet Couns. 2015;24(6):961-970. doi:10.1007/s10897-015-9836-6 25833335 PMC4592683

[7] Butrick M, Kelly S, Peshkin BN, . Disparities in uptake of BRCA1/2 genetic testing in a randomized trial of telephone counseling. Genet Med. 2015;17(6):467-475. doi:10.1038/gim.2014.125 25232856 PMC4364924

[8] Schwartz MD, Valdimarsdottir HB, Peshkin BN, . Randomized noninferiority trial of telephone versus in-person genetic counseling for hereditary breast and ovarian cancer. J Clin Oncol. 2014;32(7):618-626. doi:10.1200/JCO.2013.51.3226 24449235 PMC3927731

[9] Peshkin BN, Kelly S, Nusbaum RH, . Patient Perceptions of telephone vs in-person BRCA1/BRCA2 genetic counseling. J Genet Couns. 2016;25(3):472-482. doi:10.1007/s10897-015-9897-6 26455498 PMC4829475

[10] Jacobs AS, Schwartz MD, Valdimarsdottir H, . Patient and genetic counselor perceptions of in-person versus telephone genetic counseling for hereditary breast/ovarian cancer. Fam Cancer. 2016;15(4):529-539. doi:10.1007/s10689-016-9900-x 26969308 PMC5011450

[11] Cox A, Lucas G, Marcu A, . Cancer survivors’ experience with telehealth: a systematic review and thematic synthesis. J Med Internet Res. 2017;19(1):e11. doi:10.2196/jmir.6575 28069561 PMC5259589

[12] Lleras de Frutos M, Medina JC, Vives J, . Video conference vs face-to-face group psychotherapy for distressed cancer survivors: a randomized controlled trial. Psychooncology. 2020;29(12):1995-2003. doi:10.1002/pon.5457 32618395

[13] Centers for Medicare & Medicaid Services. Medicare Telehealth Trends Report. Published online March 21, 2024. Accessed May 24, 2024. https://data.cms.gov/summary-statistics-on-use-and-payments/medicare-service-type-reports/medicare-telehealth-trends

[14] Patel KB, Alishahi Tabriz A, Turner K, . Telemedicine adoption in an NCI-designated cancer center during the COVID-19 pandemic: a report on patient experience of care. J Natl Compr Canc Netw. 2023;21(5):496-502.e6. doi:10.6004/jnccn.2023.7008 37156477 PMC10777340

[15] Boucher AA, Jewett PI, Holtan SG, Lindgren BR, Hui JYC, Blaes AH. Adult hematology/oncology patient perspectives on telemedicine highlight areas of focus for future hybrid care models. Telemed J E Health. 2023;29(5):708-716. doi:10.1089/tmj.2022.0331 36194051 PMC10171940

[16] Thomson MD, Mariani AC, Williams AR, Sutton AL, Sheppard VB. Factors associated with use of and satisfaction with telehealth by adults in rural Virginia During the COVID-19 pandemic. JAMA Netw Open. 2021;4(8):e2119530. doi:10.1001/jamanetworkopen.2021.19530 34351404 PMC8343464

[17] Berlin A, Lovas M, Truong T, . Implementation and outcomes of virtual care across a tertiary cancer center during COVID-19. JAMA Oncol. 2021;7(4):597-602. doi:10.1001/jamaoncol.2020.6982 33410867 PMC7791400

[18] Xiao K, Yeung JC, Bolger JC. The safety and acceptability of using telehealth for follow-up of patients following cancer surgery: a systematic review. Eur J Surg Oncol. 2023;49(1):9-15. doi:10.1016/j.ejso.2022.08.037 36114050 PMC9458545

[19] Grootendorst M. BERTopic: Neural topic modeling with a class-based TF-IDF procedure. arXiv. Preprint posted online March 11, 2022. Accessed July 23, 2024. doi:10.48550/arXiv.2203.05794

[20] Grootendorst M. Topic Distributions. GitHub page. Last updated July 22, 2024. Accessed May 28, 2024. https://maartengr.github.io/BERTopic/getting_started/distribution/distribution.html

[21] Jiang AQ, Sablayrolles A, Mensch A, . Mistral 7B. arXiv. Preprint posted online October 10, 2023. doi:10.48550/arXiv.2310.06825

[22] Health Resources & Services Administration. Telehealth policy changes after the COVID-19 public health emergency. December 19, 2023. Accessed May 8, 2024. https://telehealth.hhs.gov/providers/telehealth-policy/policy-changes-after-the-covid-19-public-health-emergency

[23] American Telemedicine Association. ATA Action applauds house ways and means committee for unanimously advancing a two-year extension of many critical telehealth flexibilities. American Telemedicine Association press release. May 8, 2024. Accessed May 28, 2024. https://www.americantelemed.org/press-releases/ata-action-applauds-house-ways-and-means-committee-for-unanimously-advancing-a-two-year-extension-of-many-critical-telehealth-flexibilities/

