# Supplementary material for: Perspectives on Telemedicine Visits Reported by Patients With Cancer
Source: JAMA Netw Open. 2024 Nov 15;7(11):e2445363. doi: 10.1001/jamanetworkopen.2024.45363 (PMC11568458; doi:10.1001/jamanetworkopen.2024.45363)
Supplement: Supplement 1. — eAppendix. Survey Questions eFigure 1. Topic Cloud of the Key Topics eFigure 2. Top Keywords Associated with Key Topics eTable. Key Topics, Representative Comments, and Language Modeling Summary [file jamanetwopen-e2445363-s001.pdf]

Supplemental Online Content

Doshi SD, Charvadeh YK, Seier K, et al. Perspectives on telemedicine visits reported by patients with cancer. *JAMA Netw Open.* 2024;7(11):e2445363. doi:10.1001/jamanetworkopen.2024.45363

eAppendix. Survey Questions

eFigure 1. Topic Cloud of the Key Topics

eFigure 2. Top Keywords Associated with Key Topics

eTable. Key Topics, Representative Comments, and Language Modeling Summary

This supplemental material has been provided by the authors to give readers additional information about their work.

© 2024 Doshi SD et al. *JAMA Network Open*.

eAppendix. Survey Questions

Telemedicine Visit Satisfaction Survey

Please tell us about your recent telemedicine visit with your healthcare provider. A telemedicine visit is a visit with your healthcare provider using video through your computer or phone. Our services have recently changed, so please complete this survey even if you've completed it in the past. Your answers will help us improve the services that we provide. This survey will take about 2 minutes to complete.

I was satisfied with the instructions I received about how to connect to my telemedicine visit

- Strongly agree
- Agree
- Neither agree or disagree
- Disagree
- Strongly disagree
- I did not receive written or video instructions

Comments:

I spoke with an MSK staff member about how to connect to my telemedicine visit before my appointment

- Yes
- No

Comments:

It was easy to use my device (tablet, smartphone or computer) to connect with my provider

- Strongly agree
- Agree
- Neither agree or disagree
- Disagree
- Strongly disagree

Comments:

I was satisfied with the time it took to connect to my provider

- Strongly agree
- Agree
- Neither agree or disagree
- Disagree
- Strongly disagree

Comments:

© 2024 Doshi SD et al. *JAMA Network Open*.

Which of the following features did you like using during your telemedicine visit? (Select all that apply.)

- Joining from the link in my email or text message
- Testing my video and audio before the visit
- Inviting my friends or family to join the visit
- Using the chat to send messages
- Sharing my screen
- Other
- I didn't use any of these features

Comments:

I would have another telemedicine visit with my healthcare provider

- Strongly agree
- Agree
- Neither agree or disagree
- Disagree
- Strongly disagree

Comments:

I would recommend telemedicine to other patients like me

- Strongly agree
- Agree
- Neither agree or disagree
- Disagree
- Strongly disagree

Comments:

How did having a telemedicine visit compare with having an in-person visit with your healthcare provider?

- Having a televisit was better
- Having a televisit was about the same

© 2024 Doshi SD et al. *JAMA Network Open*.

Having a televisit was not as good
Not sure

Was this your first telemedicine visit with an MSK healthcare provider?
Yes
No

What type of device did you use for today's telemedicine visit?
Android phone iPhone
Tablet iPad
Laptop computer Desktop computer Not sure

During your telemedicine visit, did your healthcare provider talk with you about taking part in a clinical trial? Clinical trials are research studies that test new treatments to see how well they work.
Yes
No

We want to better understand your experience talking with your healthcare provider about taking part in a clinical trial. Talking with a healthcare provider about whether to take part in a clinical trial is part of the informed consent process. This information will help us to improve the informed consent process for MSK patients.

Considering everything about your visit, such as time, cost, convenience, quality of care, interaction with your healthcare team, what type of appointment would you have preferred to have for the informed consent process?

Telemedicine visit

In person

No preference

Have you ever had an in-person visit at MSK?
Yes
No

We would like to learn more about your comfort with making decisions about your health during a visit with your healthcare provider.
Please tell us whether you would be more comfortable doing the following in person or through telemedicine...

Asking for more information to help you better understand a clinical trial.
More comfortable through telemedicine
No difference
More comfortable in person

Sharing a concern about taking part in a clinical trial.
More comfortable through telemedicine
No difference
More comfortable in person

Asking for more time to make your decision about taking part in a clinical trial.
More comfortable through telemedicine
No difference
More comfortable in person

Telling your healthcare team that you don’t think a clinical trial is right for you.
More comfortable through telemedicine
No difference
More comfortable in person

Looking for information online.
More comfortable through telemedicine
No difference

© 2024 Doshi SD et al. *JAMA Network Open*.

◯ More comfortable in person

Including a friend, family member, or caregiver to join the discussion about a clinical trial.

◯ More comfortable through telemedicine
◯ No difference
◯ More comfortable in person

Please share any other thoughts or feelings about the informed consent process

© 2024 Doshi SD et al. *JAMA Network Open*.

eFigure 1. Topic Cloud of the Key Topics

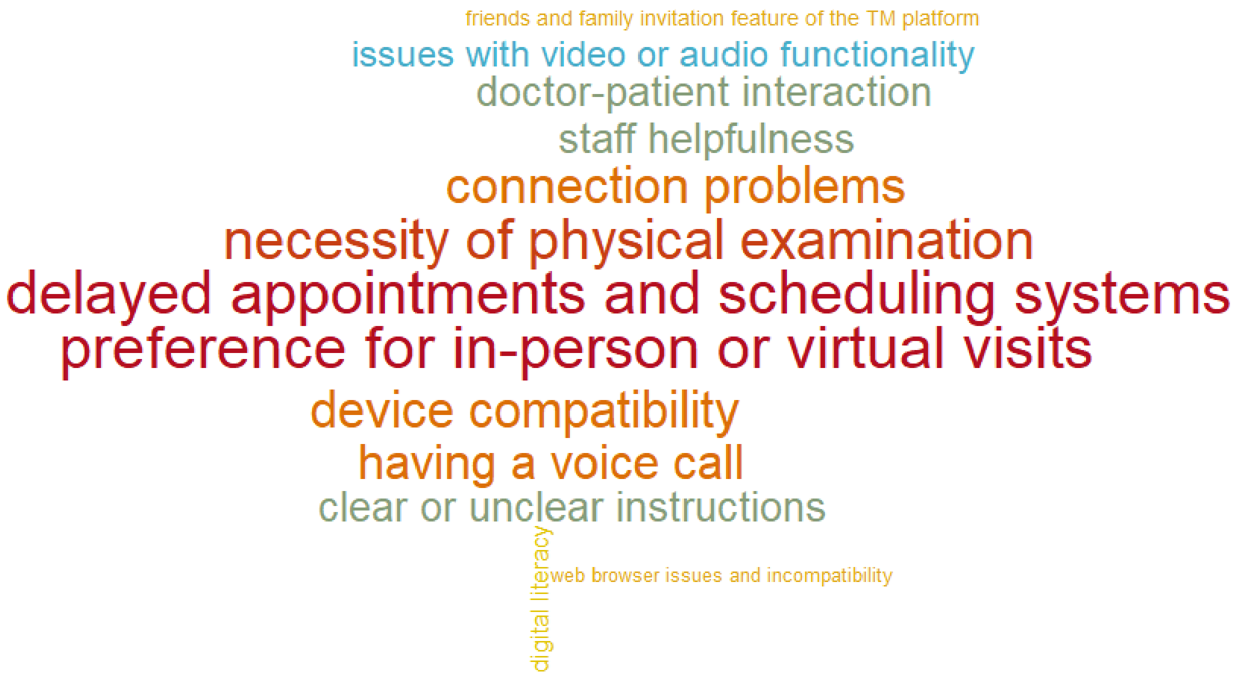

© 2024 Doshi SD et al. *JAMA Network Open.*

eFigure 2. Top Keywords Associated with Key Topics

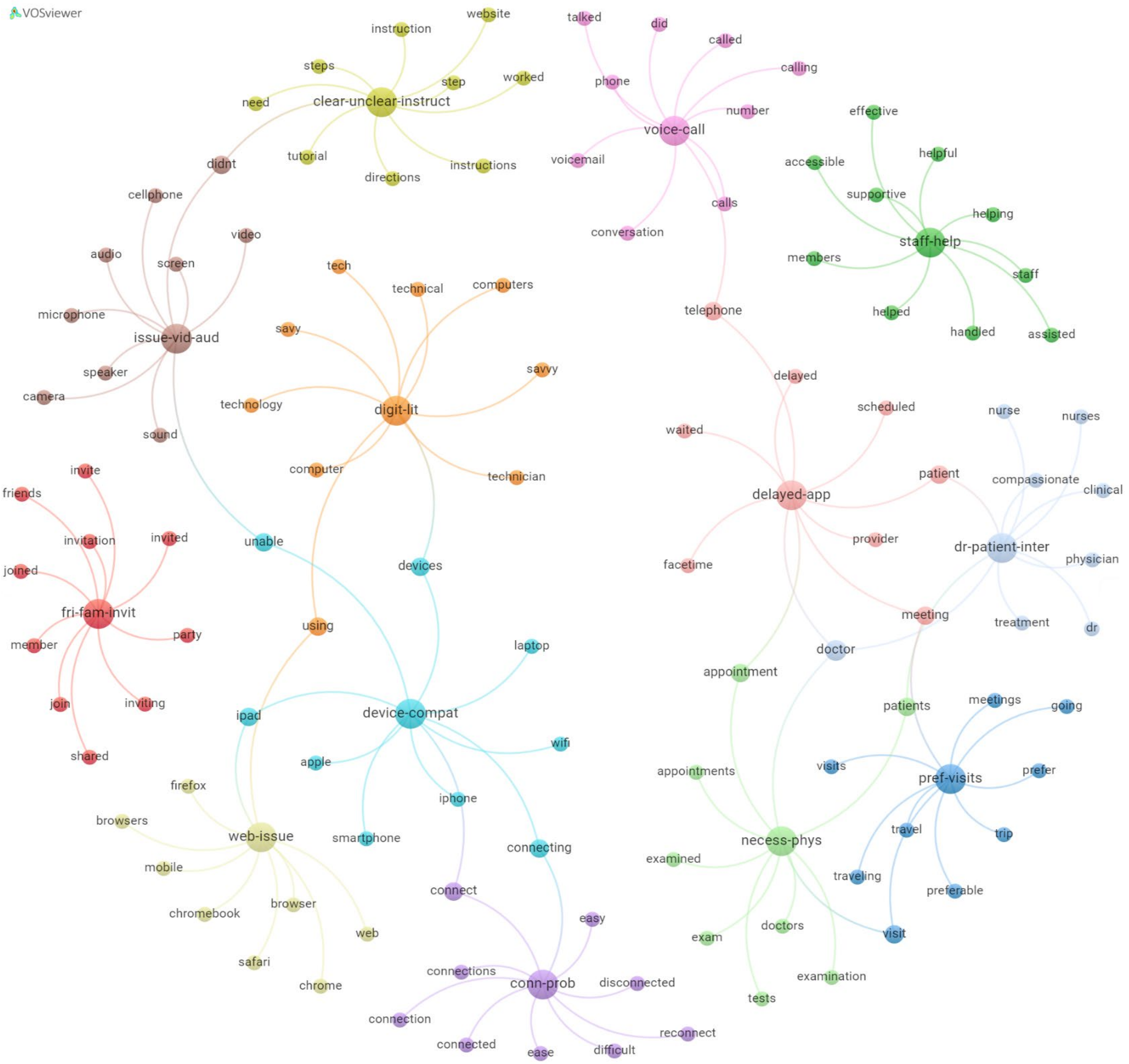

delayed-app: delayed appointments and scheduling systems; **issue-vid-aud**: issues with video or audio functionality; **pref-visits**: preference for in-person or virtual visits; **necess-phys**: necessity of physical examination; **clear-unclear-instruct**: clear or unclear instructions; **conn-prob**: connection problems; **staff-help**: staff helpfulness; **voice-call**: having a voice call; **device-compat**: device compatibility; **dr-patient-inter**: doctor-patient interaction; **web-issue**: web browser issues and incompatibility; **digit-lit**: digital literacy; **fri-fam-invit**: friends and family invitation feature of the TM platform

eTable 1. Key Topics, Representative Comments, and Language Modeling Summary

Topic Name	Example Document	LM Summary
delayed appointments and scheduling systems	"Instructions said I would be called so I was waiting for a call to then connect via my smart phone. I finally called the office 15 minutes after my scheduled appointment and was told I was in the waiting room and would be contacted. I felt it was confusing."	The majority of the patients had positive experiences with televisits, praising the convenience and ease of connecting with their providers. However, some patients had issues with the connection, technology challenges, delayed appointments, and confusion about the process. Some felt it was unclear when they should log on, and a few patients mentioned they preferred in-person visits for complex procedures or discussions. Patients generally appreciated the professional and responsive care they received during televisits, especially during appointments that couldn't be done in person. Some suggestions included clearer instructions, notifications for delayed appointments, and a more flexible scheduling system. Overall, most patients were satisfied with the televisit experience, but some would have liked improvements in terms of connection, timing, and clearer communication.
issues with video or audio functionality	"Audio was in & out. Video was good."	The comments highlight a mix of positive and negative experiences with telehealth visits, with common themes including connectivity issues, poor video and audio quality, and difficulty with the consent form. Some patients appreciated the convenience and time savings of telehealth, while others preferred in-person visits. Technical difficulties were a major frustration for many patients, with some reporting that they were unable to connect to video or audio, or that the connection dropped during the visit. Others noted confusion around the consent form and

© 2024 Doshi SD et al. *JAMA Network Open.*

		instructions for the telehealth visit, and some mentioned that they were not able to see or hear their provider during the visit. Overall, while some patients reported good experiences with telehealth, the majority expressed frustration with the technical challenges and preferred in-person visits
preference for in-person or virtual visits	"In person visit is what I would prefer."	The comments indicate that telemedicine visits have become a valuable alternative to in-person visits during the pandemic, especially for those living far from hospitals or with mobility challenges. Many appreciated the convenience of avoiding travel and waiting room visits, while others expressed a preference for in-person interactions due to the benefits of physical exams and personal connection. Some patients reported technical difficulties, while others mentioned the importance of face to face interactions for certain situations. Overall, telemedicine was seen as a useful tool for certain types of appointments and conversations, and many expressed a preference for in-person visits when feasible. The opinions emphasized the importance of considering individual health situations and circumstances when deciding between telemedicine and in-person visits.
necessity of physical examination	"I would prefer my next visit to be in person so I can take my blood work and have a physical exam."	The comments provide mixed feedback on the experience of having a televisit with healthcare providers during the COVID-19 pandemic. Some patients expressed that televisits are convenient, easy, and save time and travel expenses, especially when they do not require a physical examination. They also appreciated that some doctors were able to share information and perform certain tasks virtually. However, many patients expressed a

© 2024 Doshi SD et al. *JAMA Network Open.*

		strong preference for in-person visits and highlighted the importance of physical exams, face-to-face communication, and the emotional connection that comes with meeting their healthcare provider in person. They also noted some technical difficulties and issues with Connectivity during televisits. Overall, the key topics that emerged include the need for flexibility in choosing between televisits and in-person visits, the importance of physical exams, and the emotional and personal impact of interacting with healthcare providers in person.
Clear or unclear instructions	"Well done with the instructions."	The comments highlight a mix of experiences with televisits, with some patients expressing clear instructions and easy connections, while others faced difficulties with technology and lack of clear instructions. Many patients mentioned receiving instructions at the last minute or not receiving any instructions at all. Some found the process convenient and appreciated the savings in time and travel, while others missed the physical exam and interaction with their doctor. The most common complaints included unclear instructions, technical difficulties, and confusion about the format of the televisit (video or audio only). A few patients also suggested that written instructions should be sent in advance and that staff members were helpful and reassuring in guiding them through the process. Overall, the satisfaction level seemed to depend on the clarity of instructions and the patient's comfort level with technology.
connection problems	"It was very easy to connect."	The comments convey a mix of positive and negative experiences with connecting to telehealth visits. Some individuals reported easy and quick

© 2024 Doshi SD et al. *JAMA Network Open.*

		connections, while others encountered issues such as poor connection quality, difficulty connecting via specific devices, and lengthy wait times. Several reviewers praised the helpfulness of MSK staff in assisting them with connection issues. Instructions for connection were generally considered clear, but some individuals found them confusing or inadequate. Overall, the comments suggest that while telehealth connections can be convenient and effective, they can also present challenges that require patience and technical expertise.
staff helpfulness	"Staff was very helpful."	The majority of reviewers expressed satisfaction with their televisit experiences, praising the helpfulness and professionalism of the staff, clear instructions, and ability to connect with their healthcare providers. Many mentioned that the technology worked well and that they appreciated the convenience of the appointments, which allowed them to avoid travel and wait times. However, some reviewers reported technical difficulties and expressed frustration with the helpdesk or staff response times. Others mentioned that they preferred in-person appointments for certain procedures or exams that require physical contact. While most reviewers felt their questions and concerns were addressed, some suggested that the ability to invite family members or friends to join the call would be helpful. Overall, the comments highlight the importance of clear communication, technical support, and flexibility in delivering effective televisit appointments.

© 2024 Doshi SD et al. *JAMA Network Open.*

having a voice call	"It was a phone call."	The comments generally indicate a mix of experiences with telehealth visits, with some patients reporting successful video or audio-only visits while others encountered difficulties with connection issues or preferred traditional phone calls. Common themes include poor instructions and lack of communication from the healthcare provider's office, unclear expectations about the type of visit (video or phone), and difficulty making the connection due to technical issues or lack of familiarity with the technology. Some patients expressed preference for phone calls due to convenience or comfort, while others felt that the experience was not as thorough or personal as an in-person visit. Technical glitches, such as frozen video or poor audio quality, also contributed to negative experiences. Overall, patients appreciated the convenience of telehealth but expressed a need for clearer instructions, better communication, and more reliable technology to ensure successful visits.
device compatibility	"Couldn't connect my computer. Only iPad or phone."	The comments highlight a mix of positive and negative experiences with telehealth visits using various devices such as computers, iPads, and smartphones. Some patients had trouble connecting, encountered audio or video issues, or experienced disconnections during the visit. Others appreciated the convenience and ease of use of the technology and preferred it to in-person visits. Some patients also mentioned receiving inadequate instructions or assistance from the MSK staff in setting up the visit. Additionally, there were reports of differing experiences with specific devices or operating systems. Overall, the comments indicate that while

© 2024 Doshi SD et al. *JAMA Network Open.*

		telehealth visits offer benefits, there are also challenges and room for improvement in the experience.
doctor-patient interaction	“Dr. G. answered all our questions satisfactorially.”	The comments generally express satisfaction with the telehealth visits, with many patients praising the convenience and thoroughness of the appointments. Some patients had difficulties with the technology, such as connection issues or trouble hearing or seeing the doctor. Others expressed a preference for in-person visits due to the lack of physical examinations or the impersonal nature of the visits. Several patients highlighted the kindness and professionalism of the staff and doctors they interacted with. Some also commented on the timeliness of the appointments, with some doctors running late and others starting on time. Overall, the comments show a mix of positive and negative experiences, with most patients expressing gratitude for the ability to see their doctors during the pandemic.
web browser issues and incompatibility	“I was not told that you needed to use chrome as your browser.”	The comments highlight varying experiences with MSKCC's telehealth platform. Many users had trouble connecting due to browser compatibility issues, particularly with Safari, and had to download additional software or use different devices to attend their appointments. Some were not informed in advance about the need to use specific browsers and encountered unexpected issues during their visits. Additionally, poor audio and video quality, lack of support for certain features, and difficulty getting help from technical support were common complaints. Overall, users recommended improving the user experience and providing clearer instructions to avoid the frustration and

© 2024 Doshi SD et al. *JAMA Network Open.*

		confusion they encountered during their telehealth visits.
digital literacy	“If you are not tech savvy you might have trouble.”	The comments reflect a mix of experiences with technical difficulties and successful telemedicine visits. Some patients encountered issues with logging in, audio and video connectivity, and connecting to their healthcare providers. They noted the need for clearer instructions and more prompt technical support. Others praised the convenience and quality of the virtual visits, particularly those who had difficulty traveling or had mobility issues. The technology used varied from computers, tablets, and smartphones, with some reporting issues with specific devices or browsers. Overall, the comments highlight the importance of effective communication and technical assistance in ensuring positive patient experiences with telemedicine.
friends and family invitation feature of the TM platform	“I liked having the option to invite family or friends.”	The comments express a mix of positive and negative experiences with virtual medical visits, with the primary benefits being the convenience and elimination of travel, especially during the pandemic. Participants appreciate the ability to invite family members or friends to join the visit for added support and clarification of information. The option to use a landline phone is a common backup solution for those with technical difficulties or older devices. Some users experienced issues with the connection process and video quality, which affected their ability to fully participate or engage with their healthcare providers. Others mention that not receiving prior communication or clear instructions about the virtual visit resulted in confusion or frustration. Overall, the feedback emphasizes the

© 2024 Doshi SD et al. *JAMA Network Open.*

	importance of clear communication, accessible technology, and the flexibility to include family members or friends in the virtual visit experience.

© 2024 Doshi SD et al. *JAMA Network Open.*
