# Supplementary material for: Perspectives on Telemedicine Visits Reported by Patients With Cancer
Source: JAMA Netw Open. 2024 Nov 15;7(11):e2445363. doi: 10.1001/jamanetworkopen.2024.45363 (PMC11568458; doi:10.1001/jamanetworkopen.2024.45363)
Supplement: Supplement 2. — Data Sharing Statement [file jamanetwopen-e2445363-s002.pdf]

Data Sharing Statement

Data

Data available: Yes
Data types: Deidentified participant data
How to access data: The data will be available by contacting Dr. Sahil Doshi (doshis@mskcc.org) and/or Dr. Katherine Panageas (panageak@mskcc.org)
When available: With publication

Supporting Documents

Document types: None

Additional Information

Who can access the data: To researchers whose proposed use of the data has been approved.
Types of analyses: After review.
Mechanisms of data availability: With a signed data access agreement.
